# Training in Honey Bee Veterinary Medicine in Italy: An Observational Study and Practical Proposals to Face Professional Challenges

**DOI:** 10.3390/ani13111795

**Published:** 2023-05-29

**Authors:** Carlo D’Ascenzi, Karen Power, Paola Maiolino, Michele Mortarino

**Affiliations:** 1 Department of Veterinary Sciences, University of Pisa, 56124 Pisa, Italy; carlo.dascenzi@unipi.it; 2 Department of Veterinary Medicine and Animal Sciences, University of Milan, 26900 Lodi, Italy; 3 Department of Veterinary Medicine and Animal Productions, University of Naples “Federico II”, 80137 Naples, Italy; karen.power@unina.it (K.P.); maiolino@unina.it (P.M.); 4 Veterinary Scientific Society for Beekeeping—SVETAP, 00178 Rome, Italy

**Keywords:** honey bee, beekeeping, veterinary medicine, infectious, parasitic and abiotic diseases, food safety, Italian survey, undergraduate education, postgraduate education, veterinary specialization, roadmap

## Abstract

**Simple Summary:**

Currently, there are not many opportunities for undergraduate students to learn about Honey Bee Veterinary Medicine (HBVM) in their regular degree programs. This is a problem because veterinarians are increasingly responsible for taking care of honey bee health and production. Additionally, there are not many options for veterinarians to specialize in HBVM after graduation. This study aimed to monitor the educational opportunities for veterinarians in Italy. The study looked at both undergraduate and postgraduate programs, including degree programs in Veterinary Medicine, Masters, and other postgraduate courses. The results show that the training available for veterinarians in beekeeping in Italy is insufficient, which is a problem in other European and non-European countries as well. Finally, this study suggests a plan for training veterinarians in beekeeping that will meet the needs of the profession in the future while also following current and future public health regulations.

**Abstract:**

Honey bees, like other livestock, may be affected by infectious, parasitic, and abiotic diseases that need proper sanitary monitoring and control. Currently, there are limited opportunities for undergraduate students to receive education in Honey Bee Veterinary Medicine (HBVM) as part of their regular degree program, despite the professional requirements for veterinarians to carry out the increasing tasks related to honey bee health and production. Additionally, postgraduate training and specialization in HBVM is also underdeveloped. This study was an observational survey that evaluated the educational opportunities available in HBVM for current and future veterinarians in Italy. The survey analyzed both undergraduate and postgraduate programs, including Undergraduate Degree Programs in Veterinary Medicine (UDPVM), “Scuole di Specializzazione”, Masters, and other postgraduate courses. The results indicate that the current training available for veterinarians in the field of apiculture, both before and after graduation, is also insufficient in Italy, as already reported in other EU- and extra-EU countries. Finally, a roadmap for veterinary training in HBVM is developed here describing objectives and teachings aimed at fulfilling the needs of the profession in the field of beekeeping, considering the existing rules and regulations governing public health and possible evolution of this legal framework in the future.

## 1. Introduction

Most pollinating insects are wild, but some species are farmed for their economic value. Among the farmed species, the most numerous are those pertaining to the genus *Apis* and of which the most common is the honey bee (*Apis mellifera* L.), the third most important livestock species in Italy (97,938 farms), after cattle (134,298 farms) and small ruminant farming (135,591 farms) [1]; 650,000 beekeepers are found throughout the European Union, managing 17.5 million hives and producing about 280,000 tons of honey annually [2]. In Italy, 71,104 beekeepers have been counted, of which 51,483 produce for self-consumption (72% of the total) with about 354,802 hives (24% of the total), and 19,621 are professional beekeepers who produce for the market (28% of the total), with over 1,127,958 hives (76% of the total) [1]. Honey bees are mainly farmed for honey production; however, many others are the hive products, which have been used since ancient times in nutrition and for human psychophysical well-being [3,4,5]. The beginning of the human–honey bee relationship can be dated to at least 10,000 years ago, as documented by the rock paintings discovered in the Cave of the Spider [6]. Since then, honey bees have accompanied mankind through the centuries, and signs of the use of honey bee products have been found in all the main civilizations from Babylonians to Greeks and Romans [7,8,9].

Honey bees, together with wild species, provide about 70 percent of the pollination of all plant species on the planet, generating significant induced economic value related to the spontaneous or controlled service of pollination [10,11]. According to the Third Report on the State of Natural Capital in Italy [12] the economic value of the pollination service of Italian agricultural areas is estimated to be around 2 billion euros per year. Bees and other pollinators, such as bumblebees, represent the core of the One Health Concept, now more than ever of great interest and sensitivity, helping to maintain the balance between “Human-Animal-Environmental” Health [13]. In the One Health approach, it is increasingly evident that the beekeeping sector needs to make use of adequately trained veterinarians, both at the level of the Public Veterinary Services of Health Authorities (control activities) and at the level of the Universities (diagnostic and research activities), as well as within the veterinary freelance profession, to face the challenges represented by the monitoring, prevention, control, and eradication of notifiable diseases, as well as assuring food safety of beekeeping products.

To date, it appears that the relationship between veterinarians and beekeepers is mainly restricted to a controller–controlled relationship aimed at evaluating the compliance to regulations. On the contrary, veterinarians should support beekeepers in the implementation of national and international regulations concerning the identification, traceability, and movement of bees, following the evolution of the regulatory sector where particular emphasis is placed on the role of veterinarians in assessing the epidemiological risk connected to beekeeping, managing the use of veterinary drugs, and protecting consumers, i.e., against fraud, managing of information systems [14,15,16,17]. Under current regulations, veterinarians are called to play a key role in guaranteeing the health of honey bee colonies and of human individuals by securing the food safety of beekeeping products [18]. In addition, veterinarians should be trained to support the diverse services that beekeeping can provide, e.g., pollination of plant crops, monitoring of environmental contaminants, apitherapy, and apitourism. Finally, veterinarians should recognize and support the socio-cultural value of beekeeping, in relation to the appreciation of the natural environment, the creation of leisure, and work opportunities [19]. However, in the past decades, this field has elicited little interest among veterinarians, leading to the impoverishment of their knowledge. In order to face the new challenges in this sector, veterinarians should acquire adequate knowledge and skills during the Undergraduate Degree Program in Veterinary Medicine (UDPVM) to carry out the clinical examination of honey bee colonies and other veterinary tasks related to Honey Bee Veterinary Medicine (HBVM).

In this context, the interest of various institutions and organizations, both nationally and internationally, in the topic of veterinarian training in beekeeping is increasing [2,20]. Recent surveys have shown that there is considerable dissimilarity regarding the level and mode of teaching of HBVM in the different curricula of Veterinary Medicine in Europe and the rest of the world. HBVM is an area of Veterinary Medicine for which few educational opportunities are made available to undergraduate students during their regular degree program, even though as veterinarians they should have the theoretical and practical knowledge and skills that have been mentioned above. A recent study of undergraduate education in Veterinary Medicine conducted at the European Union level showed that only a portion of the UDPVM provides students with knowledge and skills in HBVM, usually as elective teaching or part of other subjects [20]. According to this study, the teaching of HBVM at the undergraduate level in the EU receives less attention in the curricula compared to other areas that are less widespread and popular, such as Veterinary Medicine of Laboratory Animals and Aquatic Species. In terms of postgraduate training, specialization in HBVM is also not particularly developed. In fact, there are only a few national specialization programs, and there Is no Veterinary College recognized by the European Board of Veterinary Specialization (EBVS) capable of providing European-wide recognition for specialists in HBVM, as is the case with other disciplines [20].

The present study was an observational survey on the educational offerings in HBVM provided by Italian universities to current and future veterinarians. The survey focused on both undergraduate and postgraduate programs analyzing the UDPVM activity in Italy, together with University Masters and Postgraduate Courses open to Veterinary Medicine graduates, and describes an educational proposal, defined y objectives and teachings, aimed at filling the needs related to the practice of the profession in the field of beekeeping.

## 2. Materials and Methods

The study analyzed the educational offerings of the thirteen Italian universities with Veterinary Medicine, namely the following: University of Messina (UNIME), University of Bari (UNIBA), University of Naples (UNINA), University of Sassari (UNISS), University of Teramo (UNITE), University of Perugia (UNIPG), University of Camerino (UNICAM), University of Pisa (UNIPI), University of Bologna (UNIBO), University of Parma (UNIPR), University of Torino (UNITO), University of Milano (UNIMI), University of Padova (UNIPD). Initially, the survey activity focused on analyzing the educational programs of the UDPVM. Data were evaluated based on the information provided by the “Universitaly” website (https://www.universitaly.it/ accessed on 15 November 2022). Particularly, for each UDPVM, the corresponding Scheda Unica Annuale (SUA) was examined, possibly supplemented by information available on the websites of the respective universities (Supplementary Table S1). For UDPVM, the source of the study curricula consists of the corresponding Manifesto Annuale degli Studi for the 2022/23 academic year or the Regolamento Didattico, to which the SUA files refer. The Manifesto Annuale degli Studi is the document which, in accordance with the academic teaching regulations, conveys the information relating to the educational offerings for the following academic year. The Regolamento Didattico, unlike the Academic Systems, is approved exclusively at the local level and specifies the organizational and functioning aspects of the UDCVM: objectives, entry requirements, subjects and other training activities, propaedeutics, curriculum, indications relating to the exams and the final exam, any attendance obligations, and other useful information. SUA is a management tool that is functional for the planning, implementation, self-assessment, and re-planning of the educational program. The survey identified the courses, which presented in the title a clear referral to HBVM subjects and in which the relative teaching commitment was quantifiable (European Credit Transfer and Accumulation System, ECTS, or hours). Teaching activities of HBVM were then classified as a separate subject incorporated into a curriculum (monographic), as part of other subjects in a curriculum (integrated), as mandatory or elective. Among these, the Settore Scientifico Disciplinare (SSD) for the UDPVM and the ECTS have also been identified and differentiated into lectures and practical lessons. The SSD corresponds to a specific academic discipline as reported in the Decreto Ministeriale, 4 October 2000 [21], and following amendments and modifications. The methodological tool consisted of the analysis, relating to the most recent academic year for which information was found, of the study curricula, on which programmatic documents are described in the list of courses with an indication of the reference SSD and any articulation into modules, as well as other training subjects. Subsequently, the survey focused on the postgraduate programs, namely “Scuole di Specializzazione”, University Masters, and “Corsi di Perfezionamento”, and a search was carried out as for the UDPVM (Table 1).

The “Scuole di Specializzazione” are Italian educational establishments regulated by the Decreto Ministeriale, 27 January 2006, n. 146 [22]. The” Scuole di Specializzazione”, which can only be accessed by graduates in Veterinary Medicine, award the title of specialist, a prerequisite for accessing professional roles in the Italian National Public Health Service. The “Corsi di Perfezionamento”, regulated by Legge 341/90 [23], are provided through post-graduate training, which allows for the development and training of high-level skills and abilities for the improvement of one’s professionalism. They are promoted by universities in collaboration with other public or private institutions, and unlike masters, do not issue an academic qualification, but a certificate of participation, which certifies the skills acquired during the course. Regarding the “Scuole di Specializzazione”, considering that for some schools the three-year course is not always activated in the same academic year, reference was made to the last three years, i.e., from 2020/21 to 2022/23. For the Masters and “Corsi di Perfezionamento”, the reference was made to the academic year 2021/22 (Table 1).

## 3. Results

The survey on UDPVM revealed that only 5/13 universities present HBVM courses in the educational curricula (UNIBA, UNINA, UNISS, UNIPI, UNIMI); particularly, 1/13 universities (UNISS) delivers mandatory, though incorporated, teaching in the field of HBVM, while 2/13 universities (UNIBA and UNINA) provide incorporated monothematic courses. Moreover, 3/13 universities (UNINA, UNIPI, UNIMI) offer a traineeship in HBVM as part of the 5th year final traineeship (Table 2).

The survey on postgraduate teaching revealed that 5/13 universities (UNINA, UNIBA, UNITE, UNIBO, UNIMI) have HBVM-dedicated teaching in the Scuole di Specializzazione, mainly as integrated courses and only 1/13 offers practical teaching (UNIMI) (Table 3).

Regarding the offering of the University Masters or “Corsi di Perfezionamento”, the survey showed that only one Master and one “Corso di Perfezionamento” are available for graduated veterinarians and are provided by UNITE and UNINA, respectively. The Master in “Beekeeping: environmental and health management” provides 30 ECTSs corresponding to 300 h of lectures, 20 ECTSs corresponding to 200 h of practical activities, 5 ECTSs of elective training activities, and 5 ECTSs for the final exam. The “Corso di Perfezionamento” in “Hygienic and sanitary management of apiaries for the protection of the environment and biodiversity” provides 15 ECTSs corresponding to a total of 375 h, of which 170 h are lectures and practical activities and 205 h are self-study.

## 4. Discussion

Despite Italian beekeeping representing one of the largest beekeeping sectors in Europe, with a significant number of apiaries and beekeepers involved, and the role that veterinarians are called to play under current regulations, the results of our survey show that Italian universities are currently unable to provide the education required. Furthermore, the lack of an appropriate teaching and training program in UDPVM is not compensated by an adequate postgraduate offering, which could fill the gaps in HBVM knowledge, leaving the education of veterinarians to personal initiative. In such a context, it appears evident the necessity to start training future veterinarians in the University by including HBVM subjects in the Day One Competencies. Day One Competencies represent the minimum standard skills required for a newly graduated veterinarian to initiate the different roles of the veterinary profession [24]. They were established by the European Coordination Committee for Veterinary Training (ECCVT) founded in 2004 by the European Association of Establishments for Veterinary Education (EAEVE), the European Board of Veterinary Specialization (EBVS), and the Federation of Veterinarians of Europe (FVE) [24]. The general basic veterinary competence is currently established by specific EU provisions [25]. A recent graduate who has achieved Day One Competencies should be able to independently perform the entry-level tasks and duties appropriate for veterinary practice and should be self-confident enough to self-practice veterinary medicine at the primary-care level, while knowing when it is appropriate to seek guidance from more experienced colleagues. Day One Competencies in HBVM are lacking in all the Universities analyzed in the present survey, and these skills should be implemented in the educational programs of UDPVM, and the educational programs should ensure that the theoretical and practical training is organized and delivered in the appropriate manner, enabling veterinarians to perform all of his or her functions soon after graduation, preparing them to face the world of work. At this juncture, the European System of Evaluation of Veterinary Training (ESEVT), developed by the EAEVE in collaboration with the FVE for the European accreditation of UDPVM, can represent the tool for the standardization of veterinary curricula in the EU and ensure the correct implementation HBVM teaching. The main tasks and fundamental skills that a veterinarian should acquire to be qualified to practice in the apiary include the clinical examination of honey bee colonies with adequate safety precautions; recognition of disease signs affecting the brood and adult honey bees; sampling with compilation of formal documentation for delivery to an authorized diagnostic laboratory; basic knowledge of laboratory tests; prevention, treatment, control, and eradication of honey bee infectious, parasitic, and abiotic diseases, including those reportable on the basis of the regulatory framework governing the sector at the national and international level; management of food safety guarantees for beehive products; and support to the beekeeper in providing services related to beekeeping and the environmental context. Special attention is required for training needs in health and food safety considering the current regulatory framework of public health and future training needs based on the evolution of guidelines and rules. Currently, the veterinary staff in charge of the official control needs skills in the beekeeping sector, which are addressed for the prevention and control of health problems; this field has been governed by consolidated national and/or international legislation based on the Regolamento di Polizia Veterinaria [26], with regard to Animal health, and on the National Residues Plan [27,28], with regard to food safety. Based on the progressive implementation of the recent regulations issued in the field of veterinary public health, the beekeeping sector is susceptible to growing interactions with the aims of the juridical assets of EU-member States, including the health and welfare of farmed animals, the quality and safety of the product, the protection of the natural and man-made environment, and the socio-economic field. The issuing of EU Regulation 2016/429 [15] provides the possibility of delegating functions, principally of official control, to self-employed veterinarians who hold the role of farm veterinarians. This regulatory innovation increases the need to recognize the validity of the knowledge and skills accumulated during the exercise of the profession, where the corresponding and recognized training courses are missing. Honey bee health care can be considered one of these areas, given that, as underlined by our study, the educational programs available during both the undergraduate and postgraduate period are often incomplete and fragmented. Consequently, the training of veterinarians in the beekeeping field is substantially based on the professional experience that can be acquired in the field while working alongside already expert colleagues and on attending a portfolio of training and updating events selected on an individual and autonomous basis. This experience may be susceptible to ex-post recognition and validation through the instrument of professional certification, which takes into consideration both passive learning and the professional activity carried out in various capacities. In any case, to date, there are no adequately structured and time-lined training courses, which can guarantee the acquisition of a satisfactory level of knowledge and skills in the beekeeping field for the veterinarian. Therefore, there is the need to establish an organic and suitably designed educational program, in terms of methods and times, which leads the veterinarian to acquire the knowledge and skills necessary for the performance of his/her duties in the beekeeping sector. For the purpose of adapting the professional profile of the veterinarian, it is possible to design a curriculum in HBVM that includes the definition of specific training objectives, the formulation of the related training commitment in terms of credits with assignment of the relevant scientific disciplinary fields, represented, e.g., by the SSD required by Italian legislation. Given the complexity of the knowledge and skills necessary for adequate preparation of the veterinarian in the beekeeping sector, the training objectives should be divided into two levels: first-level objectives to be achieved during undergraduate programs, and a series of second-level objectives to be achieved in the postgraduate courses for deepening and consolidating professional skills in HBVM. According to an ideal scenario, the former should be included in the study plan of the UDPVM, as dedicated and compulsory monothematic/monographic courses, to be formulated as shown in Table 4.

Given the foreseeable regulatory and practical difficulty of including compulsory single-subject courses in the current teaching systems, it is possible to hypothesize a more realistic scenario, according to which the first-level learning objectives can still be achieved by including the subjects reported in Table 4 into non-single-theme courses; the teaching load of such courses should, in any case, be adjusted proportionally and according to the context. The achievement of the first-level learning objectives during the UDPVM would enable the formulation of more specific second-level objectives and guarantee adequate in-depth study of the preparation of the veterinarians for the purpose of carrying out the profession in the beekeeping sector. Second-level objectives should include the following: honey bee anatomy, physiology, and immunology; feeding and nutrition; beekeeping techniques and good beekeeping practices; apicultural services, including pollination, environmental monitoring, apitherapy, and apitourism; current legislation and administrative fulfilments relating to the beekeeping registry; honey bee disease epidemiology; honey bee general pathology and anatomo-pathology; recognition, diagnosis, and treatment of the main infectious and parasitic diseases of honey bees, and related surveillance and eradication plans; impact of environmental and anthropic risk factors for the health and well-being of honey bees; honey bee pharmacosurveillance and pharmacovigilance; qualitative characterization of hive products; quality assurance and food safety of hive products. These objectives could be achieved during postgraduate training courses required for the mandatory and continuous updating of skills (Continuing Medical Education System) and similar initiatives, without necessarily relying on a specific type of training. Taken together, the above objectives should be placed more clearly among the fundamental knowledge and skills that a veterinarian should acquire in the training path. It would be desirable for EAEVE to define criteria and procedures for the inclusion of apicultural issues among the requirements that the UDPVM should possess for the purposes of European accreditation; this should also be included in the list of compulsory subjects and corresponding indicators for EAEVE assessment [24].

## 5. Conclusions

As the health and sustainability of honey bee populations become more crucial to global food security and ecosystem health, it is expected that the significance of veterinary education in beekeeping will continue to increase in the upcoming years. Based on what has been reported in recent literature and on the results of this study, the training in the field of apiculture that veterinarians may have at their disposal, both before and after graduation, cannot yet be considered sufficient. Future veterinarians need to receive education on several key topics related to beekeeping, including honey bee anatomy and physiology, behavior, diseases and pests, pesticide use and exposure, nutrition and feeding, beekeeping management practices, safety of hive-derived products, sustainable beekeeping, and research skills. The quantity and variety of teaching that should cover these subjects may vary from a necessary minimum to a more exhaustive and ideal articulation. An all-encompassing education in these subject matters will equip veterinarians to manage honey bee health effectively, prioritize sustainability, and contribute to the development of innovative solutions for beekeeping challenges. To adequately prepare veterinarians, comprehensive education in beekeeping should encompass both practical and theoretical knowledge. Finally, it would be desirable to establish a body for comparison and discussion at the international level among educational institutions active in the field of veterinary apiculture, to achieve a broad consensus on the educational objectives to be pursued and the best tools to achieve them, both in the pre-degree path and in the post-degree path.

## Figures and Tables

**Table 1:** Veterinary Medicine educational programs evaluated.

University	UDPVM	Scuole di Specializzazione		Masters and Corsi di Perfezionamento
Acronym	AY	Type	AY	AY
**UNIME**	2022/2023	Inspection of Food of Animal Origin	2021/2022 2022/2023	2021/2022
**UNIBA**	2022/2023	Inspection of Food of Animal Origin; Infectious Diseases, Prophylaxis, and Animal Health Requirements	2020/2021 2021/2022 2022/2023	2021/2022
**UNINA**	2022/2023	Inspection of Food of Animal Origin; Infectious Diseases, Prophylaxis, and Animal Health Requirements; Animal Feeding; Applied Ethology and Animal Welfare	2020/2021 2021/2022 2022/2023	2021/2022
**UNISS**	2022/2023	Inspection of Food of Animal Origin; Animal Health, Livestock Husbandry, and Productions.	2020/2021 2021/2022 2022/2023	2021/2022
**UNITE**	2022/2023	Inspection of Food of Animal Origin; Animal Health, Livestock Husbandry, and Productions	2020/2021 2021/2022 2022/2023	2021/2022
**UNIPG**	2022/2023	-	2020/2021 2021/2022 2022/2023	2021/2022
**UNICAM**	2022/2023	Animal Health, Livestock Husbandry, and Productions	2020/2021 2021/2022 2022/2023	2021/2022
**UNIPI**	2022/2023	Inspection of Food of Animal Origin; Animal Health, Livestock Husbandry, and Productions	2020/2021 2021/2022 2022/2023	2021/2022
**UNIBO**	2022/2023	Animal Health, Livestock Husbandry, and Productions	2020/2021 2021/2022 2022/2023	2021/2022
**UNIPR**	2022/2023	Inspection of Food of Animal Origin; Animal Health, Livestock Husbandry, and Productions	2020/2021 2021/2022 2022/2023	2021/2022
**UNITO**	2022/2023	Inspection of Food of Animal Origin	2020/2021 2021/2022 2022/2023	2021/2022
**UNIMI**	2022/2023	Animal Health, Livestock Husbandry, and Productions; Prophylaxis and Animal Health Requirements; Animal Feeding; Applied Ethology and Animal Welfare	2020/2021 2021/2022 2022/2023	2021/2022
**UNIPD**	2022/2023	Inspection of Food of Animal Origin; Animal Health, Livestock Husbandry, and Productions; Infectious Diseases, Prophylaxis, and Animal Health Requirements; Animal Feeding; Applied Ethology and Animal Welfare	2020/2021 2021/2022 2022/2023	2021/2022

Acronym of University, the Academic Year (AY) of Undergraduate Degree Program in Veterinary Medicine (UDPVM), “Scuola di Specializzazione”, Masters and “Corsi di Perfezionamento”, and the type of “Scuola di Specializzazione” evaluated are presented.

**Table 2:** Teaching dedicated to HBVM (5 years) in Italy.

University	Year	ECTS		Teaching		SSD
		Lectures	Practical	Type	Name	
**UNIBA**	4th	1	1	Monographic/ Mandatory	Hygiene and Safety of the Beekeeping Industry (1)	VET/04
**UNINA**	5th	3	3	Monographic/ Elective	Husbandry, Pathophysiology, and Pathology of Bees	AGR/20 VET/03
	5th	-	1	Incorporated/ Elective	Traineeship in Animal Husbandry	n.a.
**UNISS**	4th	2	-	Incorporated/ Mandatory	Inspection of Meat, Eggs, and Honey Products (2)	VET/04
**UNIPI**	4th	-	1	Incorporated/ Mandatory	Traineeship in Zooculture “Breeding of Small Species”	AGR/20
**UNIMI**	5th	-	4	Incorporated/ Elective	Traineeship in Zootechnics “Breeding of Major and Minor Species”	AGR/17

Acronym of University, Academic Year (AY) of UDPVM in which the teaching is offered, number of the European Credit Transfer and Accumulation System (ECTS) allocated for lectures and practical teaching, type of course provided, name of the teaching and Settore Scientifico Disciplinare (SSD) are presented. (1) The module is incorporated in the course “Food Safety 1”; (2) the subject is incorporated in the course “Food Inspection, Control and Certification”; n.a.: not available.

**Table 3:** Teaching dedicated to “Scuole di Specializzazione” (3 years) in Italy.

University	Year	ECTS		Type of Scuola di Specializzazione and Name of Teaching	SSD
		Lectures	Practical		
**UNIBA**	INSPECTION OF FOOD OF ANIMAL ORIGIN				
	3th	3	-	Beehive Products and Sector Legislation	VET/04
	3th	3	-	Chemical Risk Connected to Beehive Products	VET/04
**UNINA**	INSPECTION OF FOOD OF ANIMAL ORIGIN				
	1st	2	-	Inspection Of Honey, Eggs, and Derivatives and Relevant Legislation	VET/04
**UNITE**	INSPECTION OF FOOD OF ANIMAL ORIGIN				
	1st	n.a.	n.a.	Sanitary Inspection of Dairy Products, Eggs, and Honey	VET/04
**UNIBO**	ANIMAL HEALTH, LIVESTOCK HUSBANDRY, AND PRODUCTIONS				
	n.a	n.a.	n.a.	Livestock Registry Management—Sanitary Control in Beekeeping	VET/06
	INSPECTION OF FOOD OF ANIMAL ORIGIN				
	n.a	3	-	Beehive Products and Sector Legislation	VET/04
	n.a	3	-	Chemical Risk Connected to Beehive Products	VET/04
**UNIMI**	ANIMAL HEALTH, LIVESTOCK HUSBANDRY, AND PRODUCTIONS				
	2	-	1	Parasitology and Parasitic Diseases—Sanitary Management of the Apiary	VET/06

Acronym of University, Academic Year (AY) of “Scuola di Specializzazione” in which the teaching is offered, number of the European Credit Transfer and Accumulation System (ECTS) allocated for lectures and practical teaching, type of “Scuola di Specializzazione”, and name of teaching and Settore Scientifico Disciplinare (SSD) are presented. n.a.: not available.

**Table 4:** List of monographic courses aimed at achieving the first-level training objectives in HBVM in the UDPVM.

Teaching	ECTS	SSD
Honey Bee Biology and Ecology	1	AGR/02-03-11
Honey Bee Breeding and Reproduction	2	AGR/11-17-18-19-20
Pathology, Diagnosis, Therapy, and Prophylaxis of Infectious, Parasitic, and Abiotic Diseases of Honey Bees	2	VET/03-05-06-07
Commercial and Hygienic Quality of Beehive Products	2	AGR/19-VET/04
Veterinary Law and Legislation, Economics, and Management of Honey Bee Breeding	1	AGR/01-VET/08

Name of teaching, European Credit Transfer and Accumulation System (ECTS) allocated to teaching, and Settore Scientifico Disciplinare (SSD) of an ideal scenario are presented.

## Data Availability

Data are available upon request to the authors.

## Supplementary Materials

The following supporting information can be downloaded at: https://www.mdpi.com/article/10.3390/ani13111795/s1, Table S1: Web addresses of Universities and Veterinary Departments.
