# Supplementary material for: Training in Honey Bee Veterinary Medicine in Italy: An Observational Study and Practical Proposals to Face Professional Challenges
Source: Animals (Basel). 2023 May 29;13(11):1795. doi: 10.3390/ani13111795 (PMC10252088; doi:10.3390/ani13111795)
Supplement: Supplementary file 1 [file animals-13-01795-s001.zip › animals-2385873-supplementary.pdf]

Supplementary Table S1. Web addresses of Universities and Veterinary Departments

Acronym	web address	Name	web address
UNIME	https://www.unime.it/	Department of Veterinary Sciences	https://archivio.unime.it/it/dipartimenti/vet
UNIBA	https://www.uniba.it/	Department of Veterinary Medicine	https://www.uniba.it/it/ricerca/dipartimenti/dipmedveterinaria
UNINA	http://www.unina.it/	Department of Veterinary Medicine and Animal Production	https://www.mvpa-unina.org/
UNISS	https://www.uniss.it/	Department of Veterinary Medicine	https://veterinaria.uniss.it/it
UNITE	https://www.unite.it/	Department of Veterinary Medicine	https://www.unite.it/UniTE/Home/Medicina_veterinaria
UNIPG	https://www.unipg.it/	Department of Veterinary Medicine	https://medvet.unipg.it/
UNICAM	https://www.unicam.it/	School of Biosciences and Veterinary Medicine	https://veterinaria.unicam.it/it
UNIPI	https://www.unipi.it/	Department of Veterinary Sciences	https://www.vet.unipi.it/
UNIBO	https://www.unibo.it/	Department of Veterinary Medical Sciences	https://scienzemedicheveterinarie.unibo.it/
UNIPR	https://www.unipr.it/	Department of Veterinary Sciences	https://smv.unipr.it/
UNITO	https://www.unito.it/	Department of Veterinary Sciences	https://www.veterinaria.unito.it/
UNIMI	https://www.unimi.it/	Department of Veterinary Medicine and Animal Sciences	https://www.unimi.it/it/ugov/ou-structure/dipartimento-di-medicina-veterinaria-e-scienze-animali
UNIPD	https://www.unipd.it/	Department of Animal Medicine, Production and Health	https://maps.unipd.it/
